# Utility of ICG fluorescence imaging with vessel clamp for ileocecal resection while preserving ileal conduit constructed after previous total cystectomy

**DOI:** 10.1186/s40792-020-01013-6

**Published:** 2020-10-02

**Authors:** Tomoaki Okada, Kenji Kawada, Takashi Kobayashi, Toshiaki Wada, Yoshiharu Sakai

**Affiliations:** 1 Department of Surgery, Kyoto University Graduate School of Medicine, 54 Shogoin Kawahara-cho, Sakyo-ku, Kyoto, 606-8507 Japan; 2 Department of Urology, Kyoto University Graduate School of Medicine, Kyoto, Japan; 3 Department of Surgery, Kindai University Hospital, Osaka, Japan; 4 Department of Surgery, Osaka Red Cross Hospital, Osaka, Japan

**Keywords:** Colon cancer, ICG, Fluorescence imaging, Ileal conduit

## Abstract

**Background:**

Indocyanine green (ICG) is useful for evaluating the intestinal perfusion of anastomosis. Especially for patients with prior surgeries, ICG imaging enables surgeons in visualizing the anatomical field. Here, we reported the positive and negative staining techniques of ICG fluorescence with vessel clamp for determining the optimal resection area of vessels and mesentery.

**Case presentation:**

An 80-year-old man, who had an ileal conduit constructed after a prior total cystectomy, was diagnosed with ascending colon cancer. Although the tumor-feeding vessel was primarily the ileocecal artery, there was no detailed information about the blood running through the ileal conduit. At first, the ascending colon and the marginal vessels were transected at distal side of the tumor. Next, both, the ileocecal artery and the marginal artery of oral side of the ileal anastomotic site were clamped. Finally, we injected ICG intravenously to assess the blood flow. As a result, the blood flow between the ileal anastomotic site and transected ascending colon was not identified (negative staining). Therefore, we cut the root of the ileocecal artery, and dissected the peripheral mesocolon including the ileal anastomotic site. After the ileo-ascending colon anastomosis, we injected ICG intravenously again. The blood flow to the ileal conduit was preserved (positive staining).

**Conclusion:**

ICG fluorescence imaging with vessel clamp can clearly visualize the demarcation line between ischemic and non-ischemic intestinal tract. In colorectal surgeries, this technique is useful to assess the anastomotic perfusion and determine optimal dissection area of vessels and mesentery in secondary intestinal surgery.

## Introduction

In recent years, near-infrared fluorescence technology with indocyanine green (ICG) has been accepted as the most promising method that provides a real-time assessment of intestinal perfusion. In colorectal surgery, ICG fluorescence imaging is useful for evaluating the intestinal perfusion of anastomosis [1, 2]. A great advantage of ICG imaging is its capability to visualize the demarcation line between ischemic and non-ischemic intestinal tract, which helps surgeons in determining the optimal transection line.

In patients with history of previous abdominal surgeries, there can be a lack of enough anatomical information of previous surgeries, such as vessel division and intestinal transection. We have experienced the surgery of a patient with ascending colon cancer who had an ileal conduit constructed after a prior total cystectomy. Although the tumor-feeding vessel was primarily the ileocecal artery, there was no detailed information about the blood running through the ileal conduit. If the feeding artery of the ileal conduit had been unintentionally injured, the ileal conduit would have become necrotized. In this case, we hypothesized that ICG fluorescence imaging with vessel clamp could help to preserve the ileal conduit. Herein, we reported the positive and negative staining techniques of ICG fluorescence with vessel clamp for determining the optimal resection area of vessels and mesentery.

## Case presentation

**Fig. 1:**
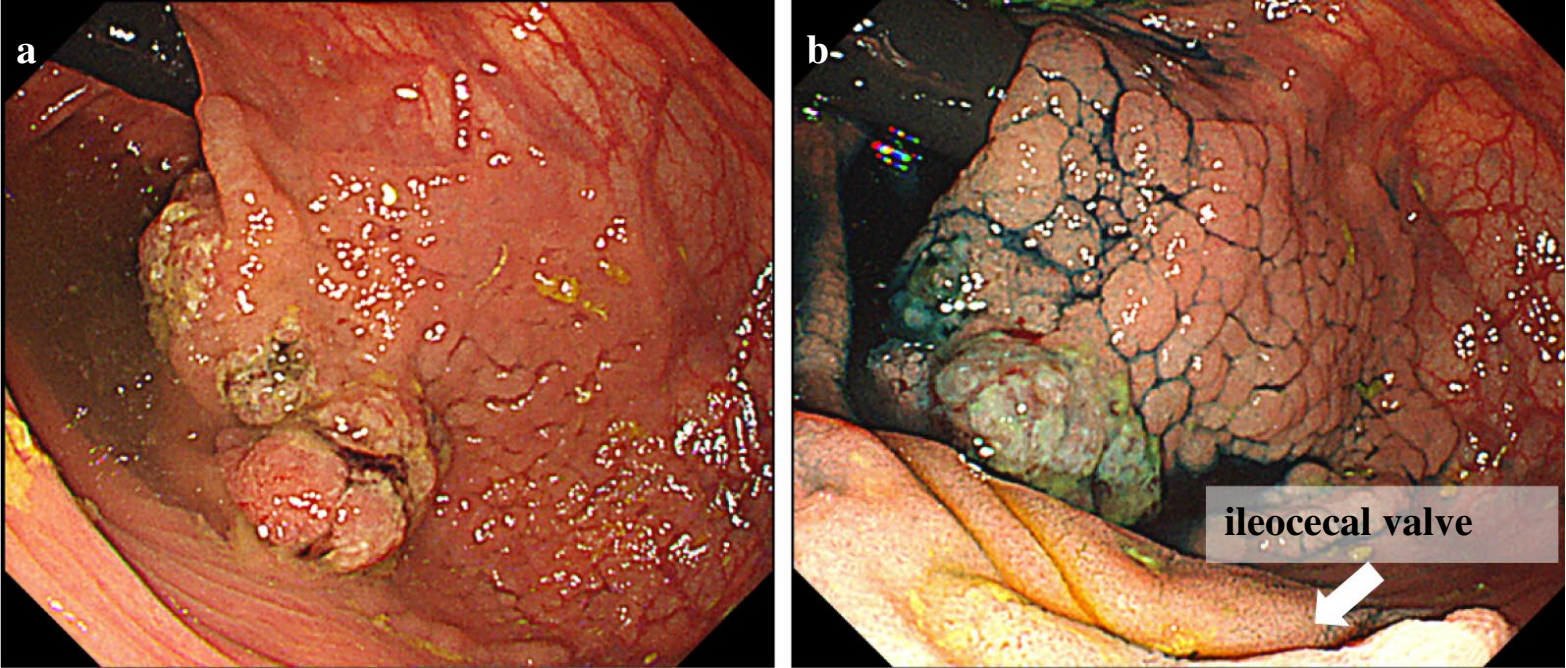
Colonoscopy images showing view from the cecum side to the ascending colon (**a**, **b**). A laterally spreading tumor is clearly visible with indigo carmine staining (**b**)

An 80-year-old man was admitted to our hospital presenting with a positive fecal occult blood test. Colonoscopy revealed that there was a laterally spreading tumor at the ascending colon near the ileocecal valve. A biopsy specimen showed an adenocarcinoma, and the depth of the tumor was suspected to be deep submucosal invasion (T1) (Fig. 1a, b). The swollen lymph nodes were not detected around the tumor by computed tomography (CT). He had undergone open total cystectomy for bladder cancer with creation of an ileal conduit at the age of 78 years. The operation record stated that the ileum 15 cm apart from the ileocecal valve was used for the ileal conduit. However, detailed information about the vessel division for the ileal conduit reconstruction was not recorded. Because the severe adhesion after open total cystectomy was expected, we selected an open approach.

## Laparotomy findings

**Fig. 2:**
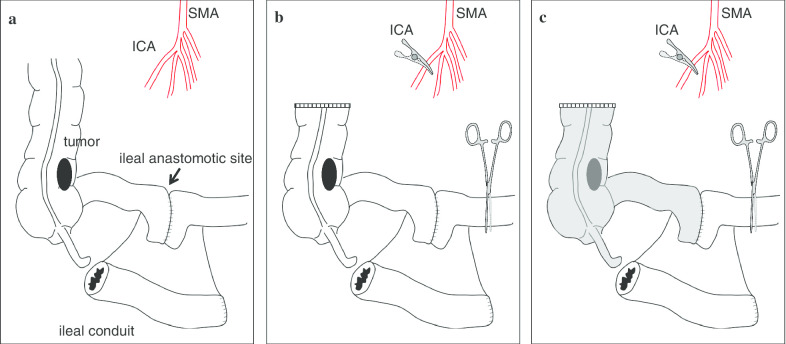
Operative schema showing relationship of the tumor, ileal anastomotic site, ileal conduit and ileocecal artery (ICA) with the superior mesenteric artery (SMA) (**a**–**c**). The ascending colon was transected, and the ICA at its root and marginal artery at oral side of ileal anastomotic site were clamped (**b**). After intravenous injection of ICG, fluorescence is clearly visible in the ischemic area (Gray color) (**c**)

After the medial incision, the right-sided colon was mobilized from the retroperitoneum. Although the ileal conduit was identified at the inferior side of the ileal anastomotic site, the vessels supplying the ileal conduit could not be identified (Fig. 2a). If the ileal conduit had been supplied from the ileocecal artery, division at its root would have resulted in the necrosis of the ileal conduit. Conversely, if the ileocecal artery and peripheral mesocolon had been preserved, the metastatic lymph nodes might have been left. We used ICG fluorescence imaging to assess the blood supply to the ileal conduit and to determine the optimal area of lymph node dissection.

## Negative staining technique of ICG fluorescence with vessel clamp

**Fig. 3:**
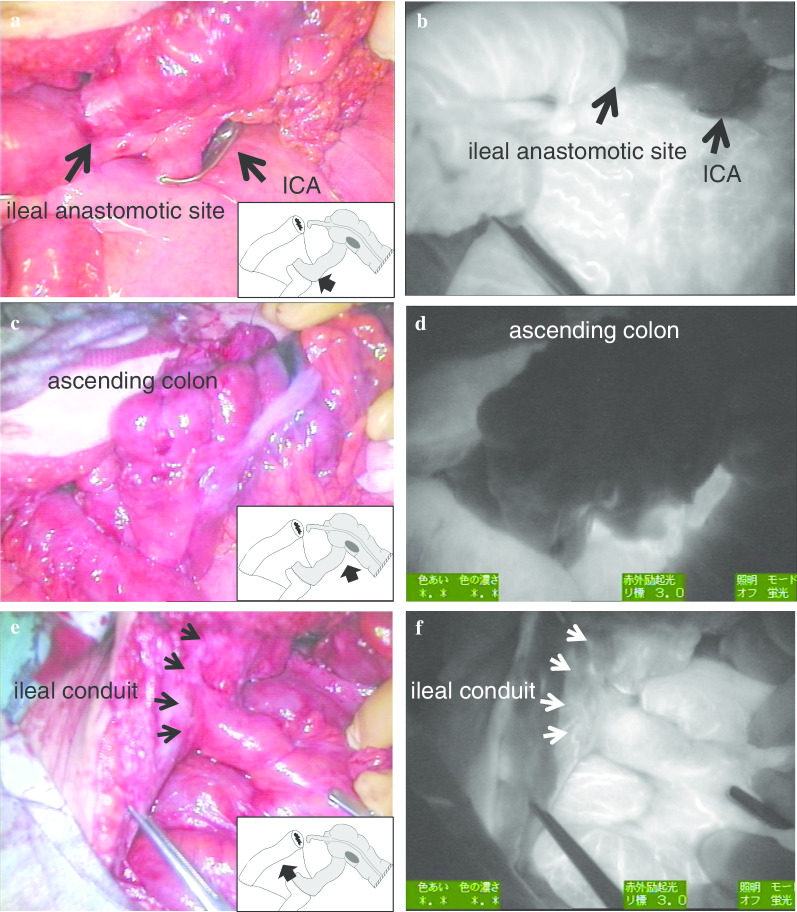
Assessment of bowel perfusion after clamping of the ileocecal artery (ICA) and marginal artery on the oral side of the ileal anastomotic site. Bowel perfusion under normal light (**a**, **c**, and **e**) and under ICG fluorescence imaging (**b**, **d**, and **f**). Intestinal segment from the ileal anastomotic site (**b**) to the ascending colon (**d**) showing a negative staining area, i.e. ischemic area. The ileal conduit showing the positive staining area, i.e. non-ischemic area (**f**)

At first, the ascending colon and the marginal vessels were transected 10 cm distal side from the tumor. Next, both, the ileocecal artery and the marginal artery of oral side of the ileal anastomotic site were clamped (Fig. 2b). Finally, we injected ICG (2.5 mg) intravenously to assess the blood flow of the ileal conduit using a near-infrared camera system (PDE-neo system; Hamamatsu Photonics, Hamamatsu, Japan). As a result, the blood flow between the ileal anastomotic site and transected ascending colon was not identified (Figs. 2c, 3a–d). However, the blood flow of the ileal conduit could be observed (Figs. 2c, 3e, f). Namely, these results indicated that the ileal conduit was supplied from the independent branch from the superior mesenteric artery, not the ileocecal artery. Therefore, we cut the root of the ileocecal artery, and dissected the peripheral mesocolon including the ileal anastomotic site. The ileo-ascending colon anastomosis was completed by functional-end-to-end anastomotic technique.

## Positive staining technique of ICG fluorescence

**Fig. 4:**
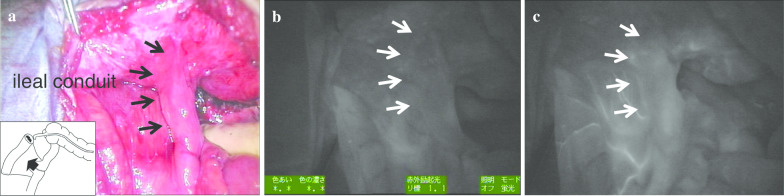
Assessment of bowel perfusion after removal of the ileocecum and ascending colon including the ileal anastomotic site. Bowel perfusion under normal light (**a**), and under ICG fluorescence imaging before (**b**) and after (**c**) intravenous injection of ICG. Brightness of the ileal conduit after intravenous re-injection of ICG (**c**) was enhanced as compared to before fluorescence (**b**)

We injected ICG (2.5 mg) intravenously again, and confirmed that the blood flow to the ileal conduit was preserved (Fig. 4a–c).

## Operative outcomes and pathological examination

**Fig. 5:**
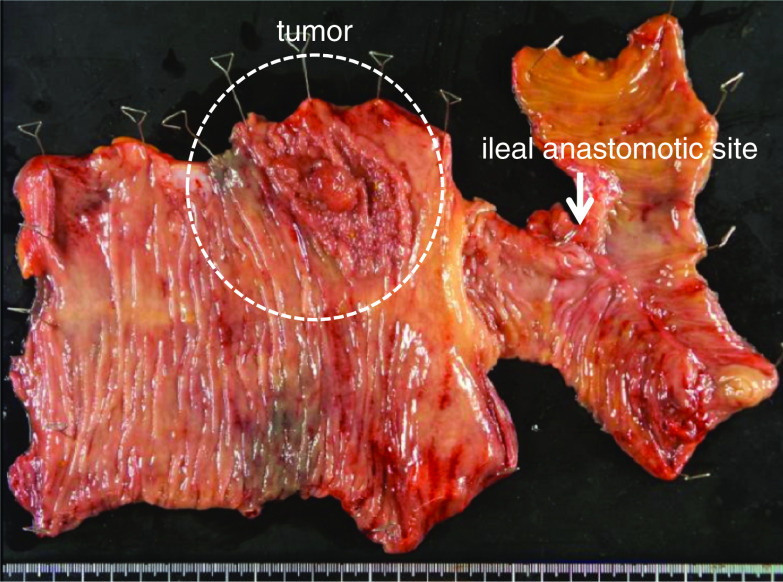
A specimen from the ileal anastomotic site to the ascending colon, including the tumor. Histological examination confirmed adenocarcinoma limited to the mucosal layer and without lymph node metastasis

Operative time was 367 min, and blood loss was 520 ml. Although mild paralytic ileus occurred on day 4 after surgery, the postoperative course was uneventful. He was discharged on the day 15 after surgery. Pathological examination indicated that the tumor was mucosal invasion (Tis) without lymph node metastasis (pN0) (Fig. 5). Nine months have passed without recurrence of colon cancer and dysfunction of the ileal conduit since surgery.

## Discussion

ICG fluorescence imaging can clearly visualize the demarcation line between ischemic and non-ischemic intestinal tract. In colorectal surgeries, this technique is useful to assess the anastomotic perfusion. Our group previously reported that the transection line was changed to the proximal side during anastomosis for left-sided colon cancer, because the ischemic area in the initially planned transection line was clearly visualized by the ICG fluorescence imaging [1]. The capability of ICG fluorescence imaging to reduce the rate of anastomotic leakage is controversial, although Watanabe et al. recently reported that ICG fluorescence imaging significantly reduced the rate of anastomotic leakage compared with control group [3]. In liver surgeries, ICG fluorescence imaging was used for navigation of hepatic segmentectomy. The demarcation line of hepatic segment can be visualized using (i) positive staining technique, in which ICG is injected directly into the portal branch of the tumor-bearing segment, and (ii) negative staining technique, in which ICG is injected intravenously with clamping the portal branch of the tumor-bearing segment [4].

In this case, there was no detailed information about the vessel division in the reconstruction of the ileal conduit. To determine the optimal resection area of vessels and mesentery, we clamped the feeding arteries to the ileocecal mesentery around the tumor including ileal anastomotic site, i.e., these areas corresponded to the Japanese D2 lymph node dissection area recommended by Japanese colorectal guidelines [5]. Only this segment was visualized as the negative staining area by ICG fluorescence imaging. Therefore, we could cut the root of the ileocecal vessels and perform D2 lymph node dissection with confidence. If the blood supply of the ileal conduit could not be observed, we would reduce the area of lymph nodes dissection taking into account the balance between the patient age and tumor invasion depth.

Additionally, we used ICG fluorescence imaging again to confirm the intestinal perfusion of the ileal conduit after removal of the tumor. This was the verification process to confirm that the feeding vessels to the ileal conduit were not unintentionally damaged during resection. When ICG is injected intravenously again, the ileal conduit could be visualized as the positive staining area.

The positive and negative staining techniques of ICG fluorescence with vessel clamp can be applied to the surgery for the metachronous colorectal cancer after primary colorectal surgery. The metachronous colorectal cancer is expected to increase gradually by the increasing of young-onset colorectal cancer [6] and prognostic improvement. This technique is useful to achieve, both, oncological curability and organ preservation.

## Conclusion

The positive and negative staining techniques of ICG fluorescence with vessel clamp was useful for determination of optimal dissection area of vessels and mesentery in secondary intestinal surgery.

## Data Availability

The datasets supporting the conclusions of this article are included within the article.

## Abbreviation

ICG: Indocyanine green

## References

[1] Kawada K, Hasegawa S, Wada T, Takahashi R, Hisamori S, Hida K, Sakai Y (2017). Evaluation of intestinal perfusion by ICG fluorescence imaging in laparoscopic colorectal surgery with DST anastomosis. Surg Endosc.

[2] Wada T, Kawada K, Takahashi R, Yoshitomi M, Hida K, Hasegawa S, Sakai Y (2017). ICG fluorescence imaging for quantitative evaluation of colonic perfusion in laparoscopic colorectal surgery. Surg Endosc.

[3] Watanabe J, Ishibe A, Suwa Y, Suwa H, Ota M, Kunisaki C (2020). Indocyanine green fluorescence imaging to reduce the risk of anastomotic leakage in laparoscopic low anterior resection for rectal cancer: a propensity score-matched cohort study. Surg Endosc.

[4] Ishizawa T, Zuker NB, Kokudo N (2012). Gayet B positive and negative staining of hepatic segments by use of fluorescent imaging techniques. Arch Surg.

[5] Hashiguchi Y, Muro K, Saito Y, Ito Y, Ajioka Y, Hamaguchi T (2020). Japanese Society for Cancer of the Colon and Rectum (JRCCR) guideline 2019 for the treatment of colorectal Cancer. Int J Clin Oncol.

[6] Mauri G, Sartore-Bianchi A, Russo AG, Marsoni S, Bardelli A (2019). Siena S Early-onset colorectal cancer in young individuals. Mol Oncol.

